# A clinical study of lung cancer dose calculation accuracy with Monte Carlo simulation

**DOI:** 10.1186/s13014-014-0287-2

**Published:** 2014-12-16

**Authors:** Yanqun Zhao, Guohai Qi, Gang Yin, Xianliang Wang, Pei Wang, Jian Li, Mingyong Xiao, Jie Li, Shengwei Kang, Xiongfei Liao

**Affiliations:** Department of Radiation Oncology, Sichuan Provincial Cancer Hospital, Chengdu, Sichuan 610041 China

**Keywords:** 3-Dimensional conformal radiation therapy, Collapsed cone convolution, Pencil beam convolution, Lung cancer, Monte Carlo, Intensity-modulated radiation therapy

## Abstract

**Background:**

The accuracy of dose calculation is crucial to the quality of treatment planning and, consequently, to the dose delivered to patients undergoing radiation therapy. Current general calculation algorithms such as Pencil Beam Convolution (PBC) and Collapsed Cone Convolution (CCC) have shortcomings in regard to severe inhomogeneities, particularly in those regions where charged particle equilibrium does not hold. The aim of this study was to evaluate the accuracy of the PBC and CCC algorithms in lung cancer radiotherapy using Monte Carlo (MC) technology.

**Methods and materials:**

Four treatment plans were designed using Oncentra Masterplan TPS for each patient. Two intensity-modulated radiation therapy (IMRT) plans were developed using the PBC and CCC algorithms, and two three-dimensional conformal therapy (3DCRT) plans were developed using the PBC and CCC algorithms. The DICOM-RT files of the treatment plans were exported to the Monte Carlo system to recalculate. The dose distributions of GTV, PTV and ipsilateral lung calculated by the TPS and MC were compared.

**Result:**

For 3DCRT and IMRT plans, the mean dose differences for GTV between the CCC and MC increased with decreasing of the GTV volume. For IMRT, the mean dose differences were found to be higher than that of 3DCRT. The CCC algorithm overestimated the GTV mean dose by approximately 3% for IMRT. For 3DCRT plans, when the volume of the GTV was greater than 100 cm^3^, the mean doses calculated by CCC and MC almost have no difference. PBC shows large deviations from the MC algorithm. For the dose to the ipsilateral lung, the CCC algorithm overestimated the dose to the entire lung, and the PBC algorithm overestimated V_20_ but underestimated V_5_; the difference in V_10_ was not statistically significant.

**Conclusions:**

PBC substantially overestimates the dose to the tumour, but the CCC is similar to the MC simulation. It is recommended that the treatment plans for lung cancer be developed using an advanced dose calculation algorithm other than PBC. MC can accurately calculate the dose distribution in lung cancer and can provide a notably effective tool for benchmarking the performance of other dose calculation algorithms within patients.

## Introduction

The accuracy of dose calculation is crucial to the quality of treatment planning and, consequently, to the dose delivered to patients undergoing radiation therapy [1]. In the past 20 years, radiotherapy has become increasingly complex. Complex treatments such as intensity-modulated radiation therapy (IMRT) are expected to provide better treatment outcomes for patients and better sparing of healthy tissues [2]. The increased complexity of the delivery and dosimetry of radiotherapy treatments arising from the increasing use of IMRT treatments has led to an increased demand for accurate treatment verification [2]. The International Commission on Radiation Units and Measurements (ICRU) recommends that patient-specific independent treatment plan verification should be performed for all IMRT treatments [3].

There is general agreement that, in IMRT, the actual delivered dose (or location) should be within 3% (or 3 mm) of the planned TPS [4]. But what is the actual dose? We need to know if the plan delivered is an accurate representation of the calculated plan. Patient-specific pre-treatment quality assurance can be performed using film, ion chamber and diode arrays. But most of Patient-specific QA were performed on phantom, which will lead to a lack of predictive power for “clinically relevant patient dose errors.” [5] The ICRU Report 83 recommends that as an alternative to a set of measured absorbed-dose distributions, it is acceptable to use independent absorbed-dose calculations instead of performing measurements. Currently, independent absorbed-dose calculations have been performed in many radiotherapy centres, but some of them use simple correction-based dose verification calculations, which are known to be less accurate than the treatment planning algorithms that they are designed to verify [6]. The use of inferior standards for QA procedures might lead to high false-negative or false-positive rates. Therefore, the accuracy of the independent absorbed-dose calculation which was used to evaluate the IMRT algorithm should be equivalent or higher than that of the treatment-planning system. A Monte Carlo algorithm would be acceptable for determining the absorbed dose in the presence of inhomogeneous tissue if the Monte Carlo code is tested sufficiently [3].

In routine clinical applications, calculations of dose to the tumour are performed by commercial treatment planning systems (TPS). The majority of these systems employ a Pencil Beam Convolution (PBC) algorithm for dose calculation. This algorithm is commonly used in clinical practice because it is very fast, but it is widely known that PBC has shortcomings in regard to severe inhomogeneities, particularly in those regions where charged particle equilibrium does not hold. This is especially questionable for target dose calculations in lung cancer treatments. In some previous studies, the prediction of PBC deviates from the measured values by as much as 15% [7,8]. Another algorithm, the Collapsed Cone Convolution (CCC), is utilised in commercial treatment planning systems, and the CCC algorithm produces values that are closer to the measured values than the PBC algorithm [9,10] but still deviates from the measurement by more than 5% under certain circumstances [7,8,11-13].

Monte Carlo (MC) simulation has proved to be the most accurate dose calculation and is therefore used to evaluate other dose calculation algorithms [7,8,11-16]. The MC method is potentially highly accurate as it can faithfully model both photon scatter and electron transport in arbitrary materials. Although benchmarking of commercial TPS dose calculations should ideally be performed against measured dose distributions, situations can occur where measurement cannot be made with high accuracy; in such situations, the MC algorithm is used as a reference against which TPS calculations can be benchmarked [17].

Although comparisons of MC calculation with the model-based calculations were reported elsewhere [12,13], great majority of previous studies have been performed for a phantom study or a specific site, and to our knowledge few studies has been devoted to intensity modulated radiation (IMRT) for clinical lung cancer. In this study, we will compare the dose distributions for 24 lung cancer patients, including the dose distributions calculated by MC, CCC and PBC for 3DCRT and IMRT.

## Materials and methods

### Patients and treatment planning

A total of 24 lung cancer patients, who had been previously treated at our hospital, were randomly selected for this study. The tumour size and location varied from patient to patient. The treatment plans were based on the patient CT scanned in a supine position under normal free breathing conditions. Mean grass tumour volume (GTV) size was 68.9 ± 56 cm^3^ (range, 5 ~ 162 cm^3^) and mean planning target volume (PTV) size was 133.9 ± 99.2 cm^3^ (range, 21 ~ 305.7 cm^3^). The Oncentra Masterplan V4.1 treatment planning system was used for the plan dose calculation. The Oncentra Masterplan TPS employs two algorithms, the Pencil Beam Convolution (PBC) and the Collapsed Cone Convolution (CCC).

Four plans were designed for each patient, two 3DCRT plans, one that used the CCC and one that used the PBC, and two IMRT plans, one of which that used the CCC and one that used the PBC for plan optimizations and final dose calculations, respectively. To facilitate the comparison, the parameters of the two IMRT plans were the same, and the conditions of the two 3DCRT plans were identical. The calculation grid size was 0.3 cm × 0.3 cm × 0.3 cm for all plans. For IMRT plans, the minimum open field size was 4 cm^2^ and minimum MU per segment was 3 MU, and the delivery type was step-and-shoot. For all of the plans, the prescribed dose was 60 Gy/30Fx, and all plans were normalized so that 95% of PTV received ≥100% prescribed dose. Radiation treatments were delivered on an 8 MV Varian 23 EX linear accelerator equipped with a multileaf collimator (60 pairs, the minimum leaf width projection into the iso-centre Plane is 5 mm). The patient’s CT and the patient’s RTPLAN files were exported to the MC work station to recalculate the dose distribution.

### Monte Carlo calculation

This study was performed using BEAMnrc [18], DOSXYZnrc [19] and a well-commissioned in-house Monte Carlo code MCSIM [20] which came from the Fox Chase Cancer Center. MCSIM is a system based on EGS4, which accepts standard DICOM-RT files exported from a commercial treatment planning system and produces MC calculated dose distributions.

The accuracy of the Monte Carlo model for the Varian 23EX linear accelerator 8 MV photon beam employed in this investigation has been thoroughly tested. In this model, the phase-space source that has been fine-tuned was used to reproduce dose profiles and percent depth doses (PDD) in water phantom. The dose profiles curves and percent depth doses curves from Monte Carlo calculation were compared to the measurements, and they were in good agreement with measurements.

**Figure 1:**
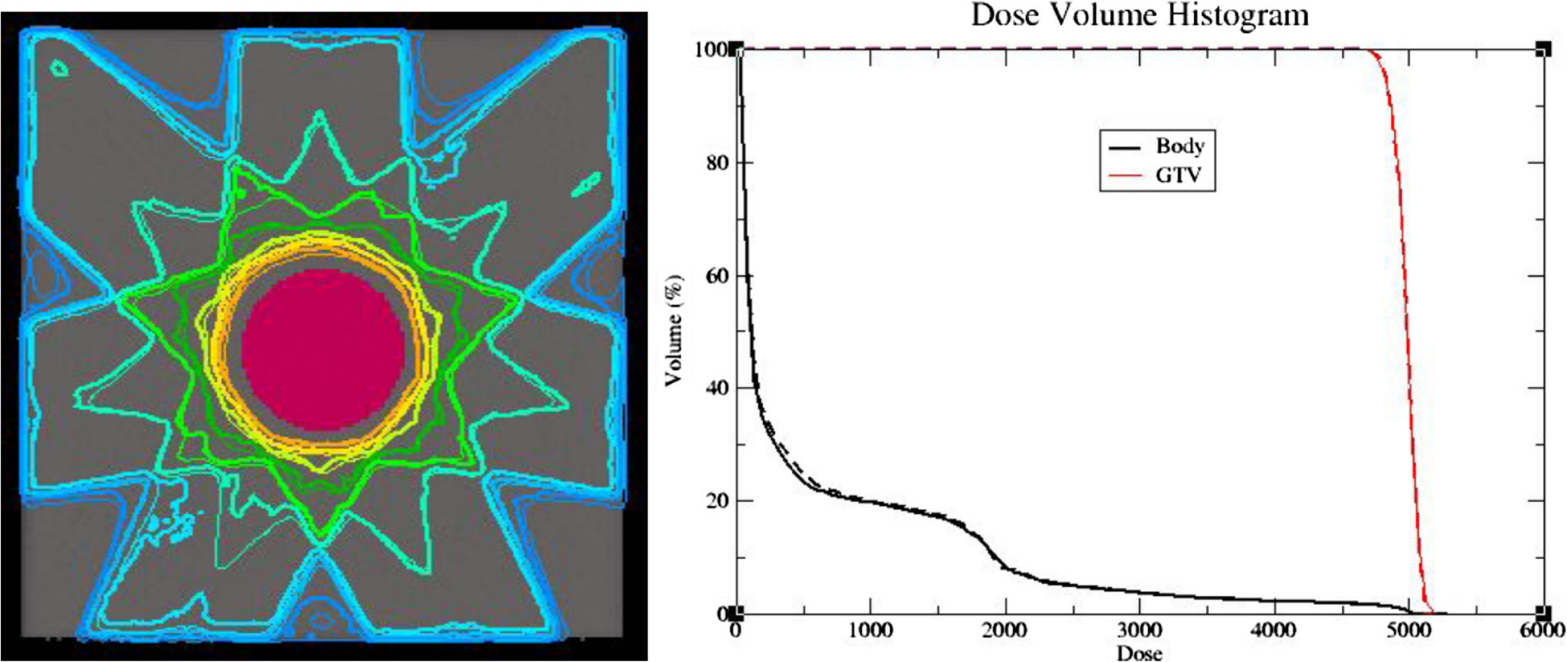
**Comparison of dose distributions and DVHs calculated using CCC and MC for a 8 cm diameter virtual tumor in solid water phantom.** On the left are the dose distributions, thick line represents MC and thin line represents TPS, On the right are the DVHs, solid line represents TPS and dotted line represents MC.

The accuracy of phase-space source also tested and verified in a solid water phantom. A solid water phantom with a virtual tumor was employed in our study, and a seven-field (10 × 10 cm^2^ open fields) 3DCRT treatment plan was created by TPS for a tumor which diameter was 8 cm. The plan was calculated using CCC and was imported in a DICOM-RT format from TPS to the MC system to recalculate. Dose distributions and cumulative dose-volume histograms (DVHs) in the water-equivalent phantom calculated by CCC and MC are shown in Figure 1. From the figure we know that the dose distributions calculated by CCC and MC agreed very well. In the same way, four 3DCRT treatment plans which field sizes were 5 × 5, 15 × 15, 20 × 20, 25 × 25 cm^2^, respectively, for corresponding virtual tumor which diameter were 3, 12, 18 and 22 cm were all recalculated using MC and compared with corresponding TPS calculations, the discrepancies in the corresponding target dose were within 1%.

In this study, the Monte Carlo calculation grid size was 0.2 cm × 0.2 cm × 0.3 cm, the ECUT = 0.7 MeV, PCUT = 0.01 MeV. In all patient calculations, we have kept the statistical uncertainty to be 2% or less so as not to significantly affect isodose lines, DVHs, or biological indices [21].

The absolute dose was calculated by converting the MC calculated dose per fluence to the dose per MU under linac calibration conditions in water (depth of 2 cm, 10 × 10 cm^2^ field size, 100 cm SSD, and 100 cGy for 100 MU).

### Lung phantom dose measurement

**Figure 2:**
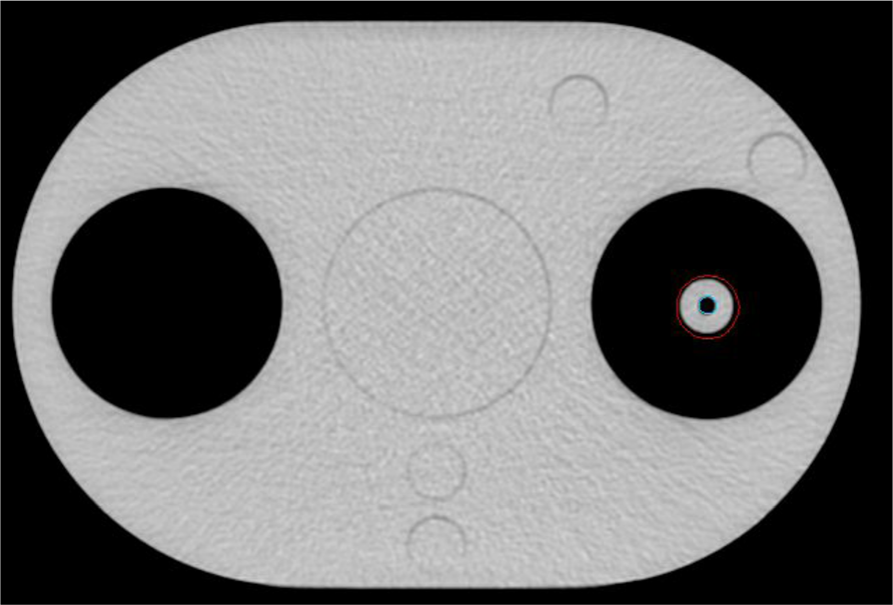
**QUASAR lung phantom with ion chamber in the tumor insert.**

The QUASAR multi-purpose body phantom was employed to model a patient thorax: it is a 30 cm wide, 12 cm long and 20 cm high acrylic body oval; with openings for cylindrical inserts of 8 cm and 2 cm diameter. These openings allow the placement of ion chambers for measurement. Two 8 cm diameter cedar wood cylindrical inserts were placed into the openings, approximately representing lung tissue, and one 2 cm diameter tumour-equivalent rod was inserted with the points of measurement. The phantom is shown in Figure 2. In the QUASAR multi-purpose body phantom, two 3DCRT plans with 5 fields were designed for the 2 cm diameter tumour using the Oncentra Masterplan treatment planning system. One plan was calculated using PBC and the other was calculated using CCC, the plan prescribed dose for 95% volume of tumour were 200 cGy. The treatment plan’s DICOM-RT files were imported into the MC system to recalculate, and the two treatment plans were also delivered on linac. An IBA CC13 ion chamber was used for dose measurement.

### Statistical analysis

The percent difference between the TPS algorithms and the MC simulations were compared for all plan evaluation parameters. Pairwise comparisons were performed using the paired t-test. P < 0.05 was considered significant. A linear regression model was used to test correlation between the percent differences in plan evaluation parameters. R^2^ values were used to evaluate goodness of fit of the linear models.

## Results

### Lung phantom dose measurement

In the QUASAR multi-purpose body phantom, the dose to the tumour centre was compared among all the calculations and measurements. The results of the TPS calculations, the MC calculations and the ion chamber measurements are listed in Table 1. The table reveals that the MC calculation doses are very close to the measurements, and the discrepancies between the MC calculations and the measurements are within 1%.

**Table 1:** **Results of dose comparison for the tumour centre among the TPS calculations and the MC simulations and measurements**

**CCC algorithm evaluation**						**PBC algorithm evaluation**					
**Meas**	**CCC**	**MUs**	**Diff**_**CCC-Meas**_	**MC**	**Diff**_**MC-Meas**_	**Meas**	**PBC**	**MUs**	**Diff**_**PBC-Meas**_	**MC**	**Diff**_**MC-Meas**_
203.1	199.6	250	−1.7%	202.6	0.2%	194.6	200.5	240	3.0%	195.8	0.6%

**Note: CCC =** Collapsed Cone Convolution; PBC = Pencil Beam Convolution; MC = Monte Carlo; Meas = measurement.

### Clinical study

Dose distributions and plan evaluation parameters calculated with the MC algorithm were compared to the PBC and CCC algorithms. The PBC and CCC result minus the MC result was expressed as a percentage. Dose-volume histograms (DVHs) were created for each patient. The GTV, PTV and ipsilateral lung were compared. Due to their clinical utility for predicting OAR toxicity, the dose-volume points were used for comparison. For lung, the dose-volume points V_5,_ V_10_, and V_20_ (the lung volume that receives at least 5, 10 and 20 Gy, respectively) were used. To evaluate the differences in the stability of the dose-volume points that denote target coverage, the dose volume points near-minimal dose (D_98_), D_95_, D_90_, D_50_, near-maximum dose (D_2_) (dose received by 98, 95, 90, 50, and 2% of the volume, respectively) were compared.

**Figure 3:**
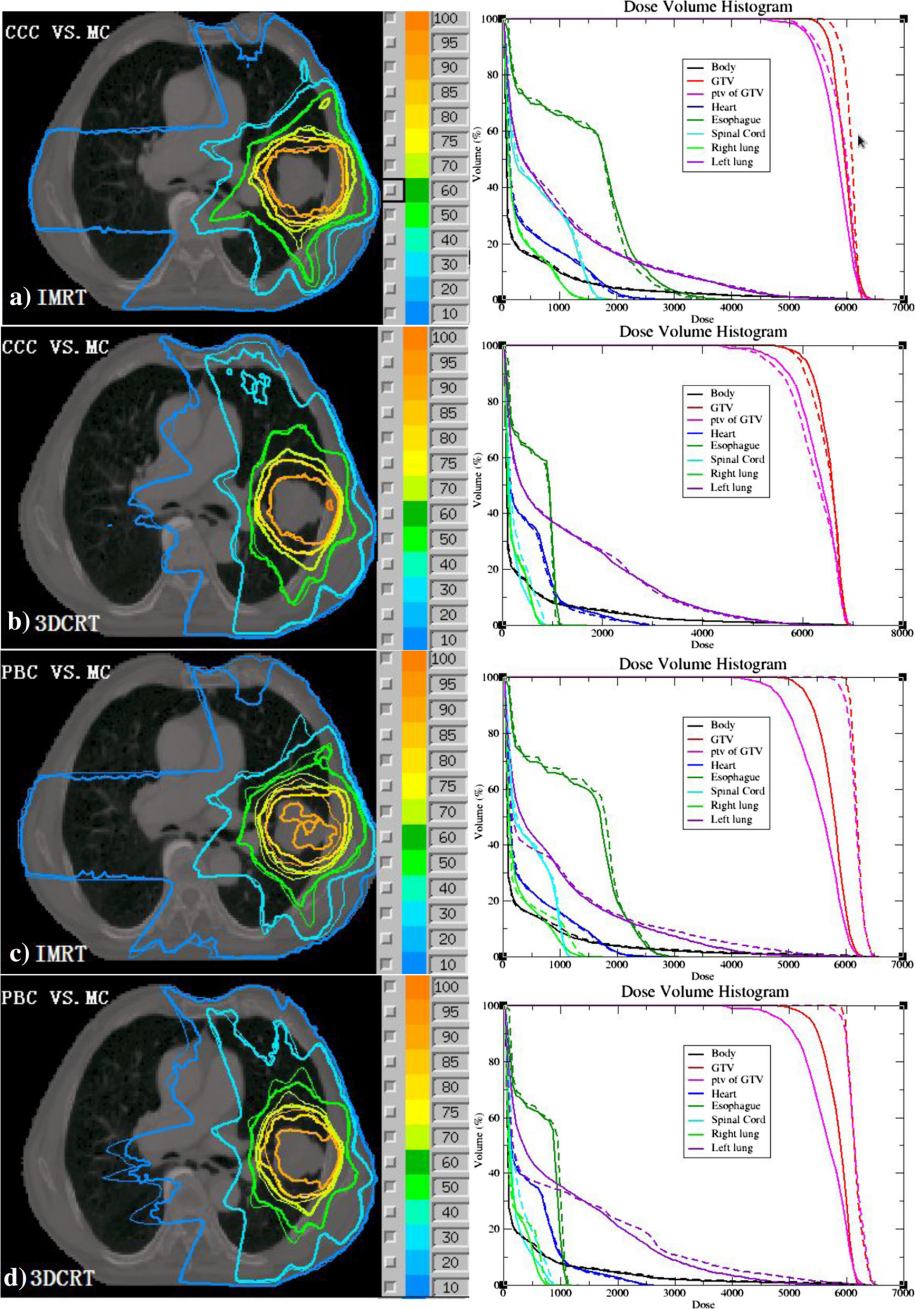
**Comparison of the dose distributions and DVHs of a lung cancer patient’s four treatment plans; on the left are the dose distributions, the thin line represents the TPS, the thick line represents MC, on the right are the DVHs corresponding to the left, dotted line represents TPS and solid line represents MC. a)** IMRT plans calculated using CCC and MC recalculation, **b)** 3DCRT plans calculated using CCC and MC recalculation **c)** IMRT plans calculated using PBC and MC recalculation **d)** 3DCRT plans calculated using PBC and MC recalculation.

Figure 3 shows the comparisons of dose distributions and DVHs of a lung cancer patient’s four treatment plans. The dose distributions are on the left and the DVHs are on the right. In general, CCC calculations are closer to MC calculations for this patient; the PBC calculations exhibit large deviations from the MC calculations.

Table 2 and Table 3 show the results of the statistical analysis of GTV, PTV and ipsilateral lung for all 24 patients, and the corresponding P values are listed in the table.

**Table 2:** **Results of the statistical analysis for GTV and PTV, for the mean dose and every dose-volume point, the results of the paired t test are reported**

			**D**_**mean**_ **(%)**	**p**	**D**_**98**_ **(%)**	**p**	**D**_**95**_ **(%)**	**p**	**D**_**90**_ **(%)**	**p**	**D**_**50**_ **(%)**	**p**	**D**_**2**_ **(%)**	**p**
3DCRT	CCC vs. MC	GTV	0.62	0.16	0.68	0.18	0.86	0.08	0.91	0.62	0.79	0.08	0.019	0.97
		PTV	0.42	0.32	−0.39	0.40	−0.25	0.61	0.05	0.91	0.53	0.22	0.013	0.98
	PBC vs. MC	GTV	5.95	0.00	7.57	0.00	7.28	0.00	6.95	0.00	5.76	0.00	4.63	0.00
		PTV	7.96	0.00	12.51	0.00	11.18	0.00	10.04	0.00	7.61	0.00	5.85	0.00
IMRT	CCC vs. MC	GTV	3.43	0.00	5.36	0.00	5.06	0.00	4.77	0.00	3.51	0.00	1.15	0.02
		PTV	3.05	0.00	3.37	0.00	3.39	0.00	3.50	0.00	3.27	0.03	2.16	0.03
	PBC vs. MC	GTV	7.81	0.00	12.84	0.00	11.49	0.00	10.50	0.00	7.44	0.00	5.04	0.00
		PTV	10.23	0.00	20.76	0.00	18.07	0.00	15.9	0.00	9.4	0.00	5.41	0.00

Note: GTV = gross tumour volume; PTV = planning target volume; MC = Monte Carlo; CCC vs. MC means (D_CCC_-D_MC_)/D_MC_; PBC vs. MC means (D_PBC_-D_MC_)/D_MC_.

**Table 3:** **Results of the statistical analysis for ipsilateral lung, results of the paired t test are reported**

		**V**_**5 Gy**_	**p**	**V**_**10 Gy**_	**p**	**V**_**20 Gy**_	**p**
3DCRT	CCC vs. MC	1.10	0.01	0.97	0.00	0.95	0.00
	PBC vs. MC	−2.70	0.00	0.17	0.47	1.49	0.00
IMRT	CCC vs. MC	1.95	0.00	1.6	0.00	1.72	0.00
	PBC vs. MC	−1.34	0.00	1.44	0.00	1.78	0.00

Note: GTV = gross tumour volume; PTV = planning target volume; MC = Monte Carlo; CCC vs. MC means V_CCC_-V_MC_; PBC vs. MC means V_PBC_-V_MC_.

**Figure 4:**
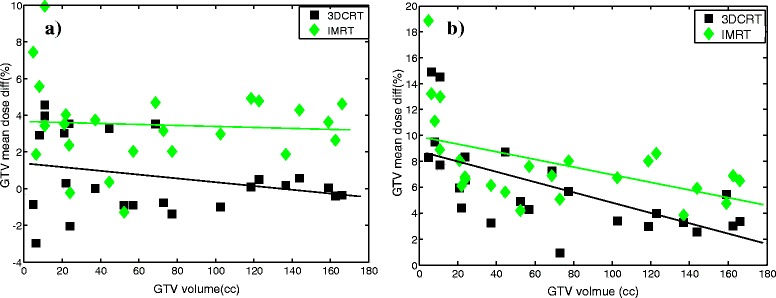
**Mean dose differences of GTV between a) CCC and MC b) PBC and MC for all individual patients.**

Figure 4a shows the mean dose differences of GTV between CCC and MC. For 3DCRT, the mean dose differences for the GTV decreases with increasing of the GTV volume; when the volume of the GTV was greater than 100 cm^3^, the mean doses calculated by CCC and MC almost have no difference. For the IMRT plans, the mean dose differences were larger than that of the 3DCRT plans. As shown in Table 2, the mean dose difference (CCC vs. MC) in the GTV for IMRT is 3.43% and 3.05% for the PTV. The mean dose difference (CCC vs. MC) in the GTV for 3DCRT is 0.62% and 0.42% for the PTV. P > 0.05 indicates that the difference between the CCC and MC for the 3DCRT plans is not statistically significant.

PBC shows large deviations from MC. Figure 4b shows that the mean dose differences for GTV between the PBC and MC increases with decreasing GTV volume for both 3DCRT and IMRT. PBC overestimated the mean dose of GTV and PTV, and when the volume of GTV was less than 10 cm^3^, the difference increases substantially, the maximum difference in the mean dose for individual patients was as high as 19%. As shown in Table 2, the mean dose difference (PBC vs. MC) in the GTV for 3DCRT plans was 5.95% and 7.96% for the PTV, and the mean dose difference (PBC vs. MC) in the GTV for IMRT plans is 7.81% and 10.23% for the PTV.

From Table 2 we know that the difference of D_98_ between CCC and MC is the largest among all dose-volume points in IMRT, and the difference become smaller gradually from D_98_、D_95_、D_90_、D_50_ to D_2_, the effect is not obvious in 3DCRT. The differences of dose-volume points for GTV between PBC and MC for all individual patients become smaller gradually from D_98_、D_95_、D_90_、D_50_ to D_2_ for IMRT plans too, the effect is less pronounced for 3DCRT. In general, the differences of dose-volume points for GTV between PBC and MC are larger than that of CCC and MC.

Table 3 shows the results of the statistical analysis for the ipsilateral lung. As shown in the table, the CCC algorithm overestimated the dose for the entire lung, overestimated V_5_, V_10_, and V_20_ by 1.1%, 0.97%, and 0.95% for the 3DCRT plans, respectively, and overestimated V_5_, V_10_, and V_20_ by 1.95%, 1.6%, and 1.72% for the IMRT plans, respectively. The PBC overestimated V_20_ by 1.49% but underestimated V_5_ by 2.7% for 3DCRT, the difference in V_10_ was not statistically significant. The PBC overestimated the average V_10_ and V_20_ by1.44% and1.78%, respectively for the IMRT plans but underestimated the average V_5_ by 1.34%.

## Discussion

In this study, we used 24 patients to investigate the effects of the dose calculation accuracy on plan evaluation for lung cancer treatments. Both based on the statistical analysis and the evaluation of all individual patient cases, it is clear that large discrepancies occur between the different dose calculation algorithms. Therefore, substantial deviations will occur when an insufficiently accurate dose computation algorithm is selected. For lung cancer treatment planning, it is very important to consider the differences in tissue density during dose calculation and to accurately model secondary electron transport [13,22]. If the lower attenuation of photon beams within lung tissue is not considered, the dose to the tissues downstream will be underestimated. Furthermore, as the electron path length within the lung extends to several centimetres, the beam penumbra widens, larger volumes of the lung are exposed to significant doses and the dose near the beam edge decreases [23,24]. Additionally, an unbalance between the number of produced and absorbed electrons arses near the interface between the low and high density tissues causes the dosage to build up [25-27]. If the electronic disequilibrium effect is not considered, it will again cause an underestimation of the dose to the lung. These effects are expected to become more pronounced for smaller field sizes, higher photon energies, and decreased lung densities [25,28,29].

The CCC is a superposition method based on a point kernel convolution with a fixed number of different directions along which the energy is transported from each grid point in the patient [17]. It employs three-dimensional (3D) density scaling of their kernels for inhomogeneity [30], whereas PBC applies only a one-dimensional convolution along fan lines [31]. The inhomogeneity correction is performed by an Equivalent Path Length correction (EPL) (i.e., using effective depths) [17].

Regarding dose reporting, PBC algorithms calculated the dose-to-water (D_w_) while the MC and CCC results reported herein were dose-to-medium (D_m_). In lung tissue and soft tissue, the differences between D_w_ and D_m_ are only approximately 1% [8,32-34]. A direct comparison of the two dose calculation algorithms (PBC and MC) is therefore possible without introducing a larger error into D_m_ vs. D_w._

Differences have been found when comparing PBC and CCC with MC algorithms for lung cancer treatment. The results of this study show that the dose prediction of CCC is closer to MC than that of PBC. Our previous study [25] and other previous studies [6-9,18] all discovered that the quantification of the difference between CCC and MC, PBC and MC depend on beam energy, lung density, target volume, target position and geometry.

In previous studies Dobler *et al.* presented the results of a comparison between PB, CC, XVMC, and film measurement in one phantom case and indicated that there was a deviation of approximately 8% between CC and measurement/XVMC for beam energy of 6MV [7]. Huixiao Chen *et al.* compared the distributions of PB, XVMC, and film measurement for typical plan applied to inhomogeneous anthropomorphic phantom, and discovered that the deviation between PB and film measurement was up to 15%. They also compared the dose calculation between PB and XVMC for 35 clinical cases, and revealed that the deviation of mean dose for PTV and GTV between PB and XVMC was approximately 7% and 4%, respectively [31]. Stephen F. Kry *et al.* retrospectively analyzed the results of 304 irradiations of the Radiological Physics Center (RPC) thorax phantom at 221 different institutions as part of credentialing for RTOG clinical trials. The results revealed that: PB algorithm overestimated the dose delivered to the centre of the target by 4.9% on average; convolution/superposition (CS) algorithms also showed a systematic overestimation of the dose to the centre of the target by 3.7% on average; in contrast, the MC algorithm dose calculations agreed with measurement within 0.6% on average [35]. For the most part, these studies are consistent with our results.

In general, the MC calculation is considered more reliable than the SC calculation. However, it should be noted that the MC calculation has an intrinsic deviation arising from statistical accuracy. This uncertainty is larger in low-dose regions.

A discrepancy between PBC and MC, CCC and MC maybe involve the uncertainty arising from both the beam modeling and CT-to-density curves used in MC calculation. MC uses the physical density as the user input, whereas Oncentra Masterplan uses the electron density.

## Conclusions

We compared the Monte Carlo algorithm with two commercial treatment planning algorithms (CCC and PBC) for 24 lung cancer patients. The CCC algorithm overestimates the mean dose to the tumour by approximately 3% in the IMRT but is very close to the MC simulation in 3DCRT. The PBC overestimates the dose to the tumour in both 3DCRT and IMRT. Therefore, it is recommended that the treatment plan for lung cancer should be calculated using an advanced dose calculation algorithm other than the PBC algorithm. In IMRT, we should be pay more attention to the minimum segment width when setting up the optimisation parameters, if the minimum segment width is too small, it will probably bring lager uncertainty. To enhance the calculation accuracy, when commissioning a treatment planning system, one should attach great importance to the similarity between the calculations and the measurements for small fields, rather than focusing on large fields [6]. In our study, the Monte Carlo dose calculation results were quite consistent with the measurements. Therefore, if the Monte Carlo code had to be benchmarked before clinical use, the MC can provide a very good tool for benchmarking the performance of other dose calculation algorithms within patients (where measurements are difficult or even impossible) [13].
